# Anatomical Study of Coronary Sinus and Thebesian Valve in Cadaveric Human Hearts

**DOI:** 10.7759/cureus.107683

**Published:** 2026-04-24

**Authors:** Urfa A Fansofkar, Punita Manik, Navneet Kumar, Sahil Srivastava

**Affiliations:** 1 Anatomy, King George's Medical University, Lucknow, IND

**Keywords:** cadaveric study, cardiac anatomy, cardiac resynchronization therapy, coronary sinus, coronary sinus cannulation, electrophysiology, thebesian valve

## Abstract

Background and objective

The coronary sinus (CS) is the principal venous channel of the heart, lying in the posterior atrioventricular groove and draining into the right atrium. At its ostium, the Thebesian valve (TV) serves as an anatomical guardian, varying considerably in morphology among individuals. These anatomical variations carry significant implications for catheter-based cardiac interventions, including CS cannulation, cardiac resynchronization therapy, and electrophysiological procedures. The objective of this study is to measure the length of the CS and to determine the prevalence and morphological types of the TV in cadaveric human hearts, as well as to assess their clinical significance.

Methods

Twenty formalin-fixed cadaveric human hearts were dissected at the Department of Anatomy, King George’s Medical University, Lucknow, India. The specimen pool comprised 12 male and eight female donors with a mean age of 42.4 ± 11.1 years (range: 21-58 years). The length of the CS was measured using precision tools, and the TV was classified into four morphological types: semilunar, remnant, cord-like, and mesh-like. High-resolution photographic documentation was performed for all specimens.

Results

The CS was present in all 20 hearts. The mean length of the CS was 44.4 ± 7.3 mm (range: 31-58 mm). Mean CS length was greater in male specimens (48.9 ± 5.5 mm) compared to female specimens (37.6 ± 3.4 mm), consistent with known sex-related differences in cardiac dimensions. The TV was identified in 90% of specimens (18 out of 20), while it was absent in 10% (two out of 20). Among the valve types, the semilunar variety was most prevalent (35%), followed by remnant (25%), cord-like (20%), and mesh-like (10%) types. Chi-square analysis revealed no statistically significant difference in TV morphological type distribution between the present study and previously published series (χ² = 6.356, df = 6, p = 0.385).

Conclusions

Significant anatomical variability exists in both CS length and TV morphology in the study population. Sex-related differences in CS length were noted and warrant further investigation in larger samples. These variations carry important implications for catheter-based cardiac procedures. The present study contributes population-specific anatomical data from the Indian subcontinent that may inform the planning and execution of interventional cardiac procedures, thereby supporting improved clinical outcomes.

## Introduction

The coronary sinus (CS) is the largest venous channel of the heart, situated in the posterior atrioventricular (AV) groove between the left atrium (LA) and the left ventricle (LV). It collects venous blood from the majority of the myocardium through its tributaries, namely the great cardiac vein (GCV), the middle cardiac vein, the small cardiac vein, the posterior vein of the LV, and the oblique vein of the LA (vein of Marshall). The CS opens into the right atrium (RA) at the inferior aspect of the interatrial septum (IAS), just superior to the AV orifice [1].

At the ostium of the CS lies the Thebesian valve (TV) (valvula sinus coronarii), a semilunar fold of endocardium first described by Adam Christian Thebesius in the eighteenth century. This valve exhibits remarkable morphological variability, ranging from a well-formed semilunar flap to a remnant tag, a cord-like fibrous structure, a mesh-like or fenestrated formation, or even complete absence [2].

With the expansion of interventional cardiology and electrophysiology (EP), the CS has become a critical structure for catheter-based procedures. These include retrograde cardioplegia, EP mapping and ablation, cardiac resynchronization therapy (CRT) involving biventricular pacemaker implantation, and LV lead placement. The TV, when prominent or structurally complex, may obstruct catheter access to the CS ostium, prolong procedural time, and increase complication risk [1,3].

Most existing cadaveric studies originate from European and Middle Eastern populations [1,2] and fewer from Indian population [3,4]. Despite the clinical significance of these anatomical variations, population-specific data from the Indian subcontinent remain limited. This variability has historically been documented in standard anatomical references, including Gray’s Anatomy (42nd Edition), which notes that the valve may exist as a thin fold, a remnant, or a net-like structure or be entirely absent [5].

The present study was undertaken to systematically document the length of the CS and the prevalence and morphological classification of the TV in cadaveric human hearts examined at King George’s Medical University (KGMU), Lucknow, India, with the aim of contributing anatomical data relevant to the Indian population and informing clinical practice.

## Materials and methods

Study design and setting

This was a descriptive observational anatomical study conducted in the Department of Anatomy, KGMU, Lucknow, India, during the academic year 2024-2025. The study followed a descriptive and comparative anatomical methodology and adhered to all institutional guidelines for cadaveric research.

Specimen selection

Twenty formalin-fixed adult human cadaveric hearts were obtained from the cadaveric dissection pool of the Department of Anatomy, KGMU. The specimen pool comprised 12 male and eight female donors. The mean age of donors was 42.4 ± 11.1 years (range: 21-58 years). Hearts with gross structural deformities, congenital anomalies, evidence of previous surgical intervention, or extensive calcification were excluded. Hearts showing advanced postmortem changes that could compromise anatomical integrity were also excluded from the study.

Dissection and measurement protocol

Each heart was systematically dissected following standard anatomical techniques. The posterior surface of the heart was exposed to visualize the CS lying in the posterior AV groove. The CS was identified by its characteristic position, size, and tributaries.

**Figure 1:**
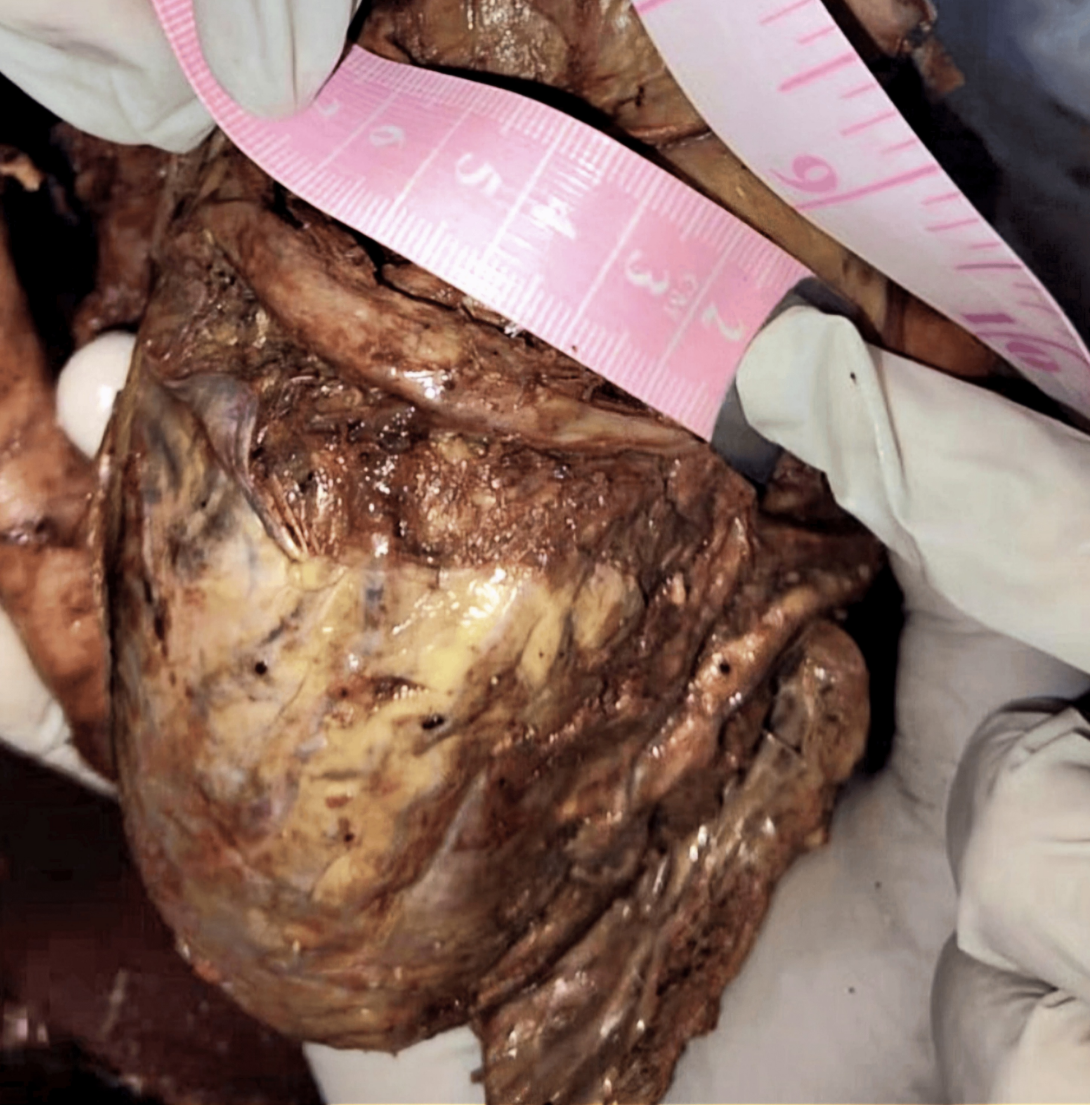
Measurement of the CS length in a cadaveric specimen using a flexible graduated scale placed along the posterior AV groove AV, atrioventricular; CS, coronary sinus

The length of the CS was measured from its commencement at the junction with the GCV, defined anatomically as the point of entry of the oblique vein of Marshall (vein of Marshall) into the GCV, which marks the transition from the GCV to the CS, to its termination at the ostium in the RA, defined as the point at which the CS opens into the right atrial cavity at the inferior aspect of the IAS, just superior to the tricuspid valve annulus. These proximal (entry of the oblique vein of Marshall) and distal landmarks (the CS ostium at the right atrial wall) are consistent with those described in Gray’s Anatomy (42nd Edition) [5] and have been used in prior cadaveric studies [1,3]. Measurements were taken along the external surface of the posterior AV groove, following the natural curvature of the CS, using a flexible graduated scale applied snugly to the groove, as represented in Figure 1.

Measurements were performed using a flexible graduated scale, with readings recorded to the nearest millimeter. All measurements were performed twice for each specimen to reduce random measurement error; the mean of the two readings was recorded. The primary investigator performed all dissections and initial measurements, and a second independent observer, trained by the same supervising faculty member, independently verified all measurements. Where discrepancies between the two observers exceeded 2 mm, the specimen was reexamined, and a consensus measurement was obtained. In practice, interobserver differences were minimal (≤1 mm in the majority of specimens), confirming acceptable reproducibility. Both observers were blinded to each other’s measurements at the time of independent recording.

The RA was opened to expose the CS ostium and identify the TV. The TV was classified into the following morphological types based on the classification described by Hołda et al. [2]: (1) semilunar type: a well-formed, crescent-shaped flap partially covering the CS ostium; (2) remnant type: a small, tag-like residual tissue at the margin of the ostium, representing a vestigial valve; (3) cord-like type: a fibrous cord traversing across or adjacent to the CS ostium; and (4) mesh-like (fenestrated) type: a net-like or sieve-like structure partially or completely covering the ostium.

In cases where no discernible valve tissue was identified at the CS ostium, the TV was classified as absent. High-resolution photographic documentation was performed for all specimens using a digital camera (Nikon D3500, 24.2 MP; Nikon Corporation, Tokyo, Japan) under standardized lighting conditions. All images were archived for reference and analysis.

Statistical analysis

Data were recorded in a structured proforma and analyzed using descriptive statistics. Continuous variables (CS length) were expressed as mean ± SD. Categorical variables (TV type) were expressed as frequencies and percentages. To compare TV morphological type distributions between the present study and previously published series [1,2], chi-square (χ²) tests of independence were performed. A p-value of <0.05 was considered statistically significant. Statistical analysis was performed using IBM SPSS Statistics for Windows, version 26.0 (released 2018; IBM Corp., Armonk, NY, USA) and Microsoft Excel 2019 (Microsoft Corporation, Redmond, WA, USA).

## Results

Specimen demographics

The specimen pool comprised 12 male (60%) and eight female (40%) donors. The mean age of all donors was 42.4 ± 11.1 years (range: 21-58 years). The mean age of male donors was 46.9 ± 8.2 years, and that of female donors was 35.6 ± 11.8 years.

Length of the CS

The CS was identified and measured in all 20 cadaveric hearts. The mean length of the CS was 44.4 ± 7.3 mm, with a range of 31 mm to 58 mm. When stratified by sex, male specimens demonstrated a mean CS length of 48.9 ± 5.5 mm, compared to 37.6 ± 3.4 mm in female specimens, consistent with established associations between cardiac dimensions and body size. Individual specimen data are presented in Table 1. These measurements are consistent with values reported in international anatomical literature and align with data from previous Indian cadaveric studies.

**Table 1:** Individual CS length measurements with TV type, sex, and age for each of the 20 cadaveric specimens CS, coronary sinus; TV, Thebesian valve

Specimen no.	Age (years)	Sex	CS length (mm)	TV type
1	21	Female	40	Absent
2	36	Male	46	Mesh-like
3	36	Male	48	Remnant
4	37	Male	49	Cord-like
5	40	Male	57	Remnant
6	44	Male	55	Semilunar
7	48	Male	46	Semilunar
8	22	Female	31	Cord-like
9	30	Female	36	Remnant
10	30	Female	42	Absent
11	49	Male	43	Mesh-like
12	41	Female	38	Semilunar
13	50	Male	53	Semilunar
14	52	Male	58	Semilunar
15	55	Male	43	Remnant
16	58	Male	43	Remnant
17	58	Male	46	Cord-like
18	43	Female	40	Cord-like
19	43	Female	36	Semilunar
20	55	Female	38	Semilunar

Prevalence and morphology of the TV

The TV was identified in 18 out of 20 specimens, yielding a prevalence of 90%. In the remaining two specimens (10%), no valve tissue was discernible at the CS ostium, and the TV was classified as absent. Among the 18 specimens with a detectable TV, the following distribution of morphological types was observed: semilunar type in seven specimens (35%), remnant type in five specimens (25%), cord-like type in four specimens (20%), and mesh-like type in two specimens (10%). These findings are summarized in Table 2.

**Table 2:** Prevalence and morphological distribution of the TV among 20 cadaveric specimens TV, Thebesian valve

TV type	No. of specimens	Percentage (%)	Status
Semilunar	7	35	Present
Remnant	5	25	Present
Cord-like	4	20	Present
Mesh-like	2	10	Present
Absent	2	10	Absent
Total	20	100	-

**Figure 2:**
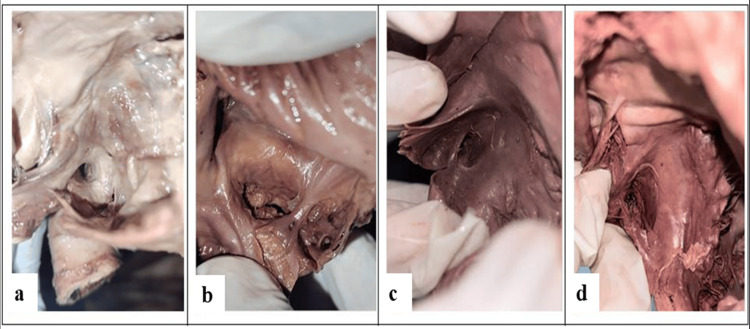
High-resolution photographs of cadaveric human hearts showing the four morphological types of the TV (a) Remnant type (25%): a small tag-like vestigial tissue at the margin of the CS ostium. (b) Semilunar type (35%): a well-formed crescent-shaped endocardial flap partially occluding the CS ostium. (c) Mesh-like type (10%): a fenestrated net-like structure spanning the CS ostium. (d) Cord-like type (20%): a fibrous cord traversing across the ostium. CS, coronary sinus; TV, Thebesian valve

The semilunar type was the most commonly observed morphological variant, consistent with findings reported in international studies. High-resolution photographs of each valve type are presented in Figure 2.

## Discussion

Length of the CS

The mean CS length recorded in the present study (44.4 ± 7.3 mm) falls comfortably within the normal range described in established anatomical references. Gray’s Anatomy (42nd Edition) cites a normal CS length of approximately 40-50 mm, and Snell’s Clinical Anatomy by Regions, 9th Edition, reports a range of 30-50 mm [5,6]. Our findings closely accord with these textbook benchmarks, providing independent corroboration from an Indian cadaveric cohort.

Comparison with prior cadaveric studies conducted in India reveals a range of reported mean values. Manoranjitham et al. [3] reported a considerably higher mean of 54.98 ± 12.2 mm in 30 specimens, while Mehra et al. [4] documented a mean CS length of 35.35 ± 4.43 mm in a series of 40 hearts. The present value of 44.4 mm lies between these two estimates, suggesting that the Indian cadaveric pool may harbor considerable inter-individual variability that is not fully captured by any single small-to-moderate sample. The international literature similarly reflects this range; Karaca et al. [1] documented a mean of 42.2 mm in 52 Turkish hearts, a figure closely comparable to our own findings. A comparative summary is presented in Table 3.

**Table 3:** Comparison of mean CS length between the present study and published cadaveric studies CS, coronary sinus

Author (year)	Sample size (n)	Mean CS length (mm)	Sex ratio (M:F)	Mean age (years)
Karaca et al. (2005) [1], Turkey	52	42.2 (average)	20:32	58 ± 14
Manoranjitham et al. (2015) [3], India	30	54.98 ± 12.2	Not reported	Not reported
Mehra et al. (2016) [4], India	40	35.35 ± 4.43	Not reported	Not reported
Present study (2026), India	20	44.4 ± 7.3	12:08	42.4 ± 11.1

It is important to note that methodological differences between studies may contribute to inter-study variability in reported CS lengths. The present study measured CS length along the external surface of the posterior AV groove using a flexible graduated scale, following the natural curvature of the structure. Manoranjitham et al. [3] and Mehra et al. [4] did not explicitly detail whether measurements were taken along the internal luminal axis or the external surface contour, nor did they specify the method of instrument positioning along the curved anatomy. Karaca et al. [1] similarly provided limited procedural detail. Such methodological heterogeneity, combined with differences in formalin fixation protocols, specimen age and sex distribution, and population body habitus, is likely to account for at least some of the observed inter-study variation in mean CS length [2]. Future studies should standardize measurement methodology to facilitate valid cross-study comparisons.

The observed sex-related difference in CS length (males 48.9 ± 5.5 mm vs. females 37.6 ± 3.4 mm) in the present study is consistent with the known association between cardiac dimensions and body surface area, with male hearts generally larger than female hearts across populations [7,8]. Hołda et al. [2], in their large Polish series of 273 hearts, found that CS ostium diameter was independent of both age and sex; however, CS length was not specifically analyzed by sex in that study. Similarly, Karaca et al. [1] reported no significant sex-related difference in CS ostium diameter in their Turkish cohort. Whether sex-related differences in CS length are a consistent anatomical finding across populations warrants dedicated investigation in larger, prospectively designed studies. Given the small sample size in the present study, the observed sex difference should be interpreted cautiously.

The wide range of CS lengths (31-58 mm in the present series) carries procedural relevance. A shorter CS restricts the depth of catheter insertion and may limit access to suitable posterolateral or lateral branch veins required for optimal LV lead placement in CRT [9]. Conversely, an unusually long CS may necessitate the use of longer sheath systems and increase the technical difficulty of catheter anchoring. Procedural planning that incorporates preoperative coronary venous imaging, such as CT venography or CS venography, is advisable in patients exhibiting anatomical extremes [10].

TV: prevalence and morphology

The TV was identified in 90% of specimens in the present study, consistent with the broadly accepted view that this structure is present in the vast majority of individuals. Its complete absence in 10% of cases aligns with the 5-15% range for TV absence reported in the literature [1,2]. These figures affirm that while the TV is a consistent anatomical landmark, its absence must always be considered during catheter-based procedures targeting the CS ostium.

Embryologically, the TV represents a vestige of the right venous valve of the sinus venosus. Incomplete regression leads to a well-formed semilunar flap, whereas more extensive regression results in the remnant or cord-like forms [11]. This developmental continuum explains the broad morphological spectrum observed across individuals and populations.

Comparison of our morphological findings with those of Hołda et al. [2], who examined 250 cadaveric hearts in Poland, and Karaca et al. [1], who studied 52 hearts in Turkey, reveals broadly concordant trends. The semilunar type was the most common form in all three studies, and the mesh-like type was the least prevalent. These findings are summarized in Table 4.

**Table 4:** Comparison of TV morphological type distribution across three cadaveric studies ^*^ Chi-square analysis comparing TV type distribution (four types: remnant, semilunar, cord-like, and mesh-like) across all three studies: χ² = 6.356, df = 6, p = 0.385. Pairwise comparisons: present study vs. Hołda et al.: χ² = 0.298, p = 0.960; present study vs. Karaca et al.: χ² = 4.330, p = 0.228; Hołda et al. vs. Karaca et al.: χ² = 5.779, p = 0.123. No statistically significant difference was found between any pair of studies or across all three studies, indicating that TV morphological type distribution in the present Indian cohort is consistent with European (Polish) and Middle Eastern (Turkish) published data. The “Absent” category was excluded from chi-square calculations as it was not reported by Karaca et al. [1] and Hołda et al. [2]. TV, Thebesian valve

Valve type	Karaca et al. (2005) [1], Turkey (n = 52)	Hołda et al. (2015) [2], Poland (n = 250)	Present study (2026), India (n = 20)	Chi-square p-value^*^ (overall = 0.385)
Remnant	45.7%	25.5%	25%	-
Semilunar	42.9%	32.6%	35%	-
Cord-like	5.7%	14.3%	20%	-
Mesh-like	5.7%	10.3%	10%	-
Absent	Not reported	Not reported	10%	-

A noteworthy finding is the comparatively higher prevalence of the cord-like TV type in the present Indian cohort (20%) versus the Polish (14.3%) and Turkish series (5.7%) [1,2]. While the small sample size precludes definitive conclusions, this disparity may reflect true population-level differences in the degree of sinus venosus valve regression. However, the chi-square test result (p = 0.228 for Present vs. Karaca) confirms that this apparent difference does not reach statistical significance and must, therefore, be interpreted as a preliminary observation requiring confirmation in larger studies. Larger multiethnic studies are warranted to determine whether this apparent difference is reproducible and clinically meaningful.

The cord-like and mesh-like TV variants are of particular clinical concern. A cord-like TV traversing the CS ostium may deflect a catheter tip and prevent successful cannulation or predispose to inadvertent perforation of the posterior RA wall during forceful advancement [1,12]. The mesh-like (fenestrated) type poses a different challenge: catheter entrapment within the fenestrated tissue has been described, increasing the risk of arrhythmia, hematoma, and valve avulsion during catheter withdrawal [2]. Awareness of these morphological possibilities is therefore of practical value for electrophysiologists and interventional cardiologists.

Limitations

The principal limitation of the present study is the small sample size (n = 20), which restricts statistical power and the generalizability of findings to the broader Indian population. Future studies with larger sample sizes and multi-institutional collaboration are warranted to establish robust population-specific normative data.

Although donor age and sex data were available for all 20 specimens in the present study (mean age 42.4 ± 11.1 years; 12 males, eight females), the sample is insufficiently powered to draw statistically robust conclusions about the effects of age or sex on CS length or TV morphology. The observed sex-related difference in CS length (males 48.9 ± 5.5 mm vs. females 37.6 ± 3.4 mm) is a preliminary observation and should not be overinterpreted; formal comparison of sex-stratified CS lengths was not the primary aim of this study, and a dedicated, adequately powered study would be required to confirm this finding.

The use of formalin-fixed specimens introduces potential postmortem tissue distortion, including shrinkage and alterations in soft tissue architecture, which may influence absolute CS length measurements. This limitation is common to all cadaveric anatomical investigations using formalin fixation and has been acknowledged in comparable studies [2,7]. The degree of distortion is unlikely to materially affect TV morphological classification but could introduce systematic measurement bias in CS length values.

Measurement reproducibility was ensured by performing each CS length measurement twice per specimen and by independent verification by a second observer trained by the same supervising faculty member. Interobserver discrepancies were ≤1 mm in the majority of cases. Despite these precautions, residual intra- and interobserver variability cannot be excluded entirely, and formal intraclass correlation coefficient analysis, which would constitute best methodological practice, was not performed in this study. Future investigations should incorporate formal reliability analyses to quantify measurement precision.

As a single-center study at a single institution in northern India, the findings have limited external validity and may not be representative of the full range of anatomical variation across the Indian subcontinent, which encompasses considerable genetic, anthropometric, and environmental diversity. The findings should therefore be interpreted as preliminary data requiring validation in larger, prospectively designed, multicenter studies.

Clinical significance

The clinical importance of CS anatomy has expanded considerably with the proliferation of catheter-based cardiac procedures. In CRT, LV pacing leads are advanced through the CS into lateral or posterolateral tributary branches to achieve biventricular synchrony in patients with heart failure (HF) and left bundle branch block [9,12]. The success of LV lead placement is directly influenced by CS ostium anatomy, CS length, and branch vessel distribution; suboptimal lead positioning is associated with attenuated CRT response and worse long-term outcomes [9,13]. Beyond CRT, the CS serves as an essential anatomical landmark during electrophysiological mapping, ablation of accessory pathways in Wolff-Parkinson-White syndrome, and cavotricuspid isthmus ablation for typical atrial flutter; a complex TV morphology can significantly delay these procedures and increase radiation exposure [12]. Retrograde cardioplegia during open cardiac surgery similarly depends on reliable CS cannulation, and an obstructive TV or tortuous CS may compromise myocardial protection [14].

The wide CS length range observed in the present study (31-58 mm) and the relatively high prevalence of cord-like and mesh-like TV types (collectively 30% of TV-positive specimens) are of procedural relevance. Preprocedural coronary venous imaging, such as CT venography or CS venography, can be advisable in patients with anticipated anatomical complexity [10], and familiarity with TV morphological variants enables the proceduralist to select appropriate instrumentation in advance.

From a population health perspective, the Indian subcontinent accounts for a disproportionate burden of cardiovascular disease, including HF and arrhythmias requiring EP intervention [15]. As interventional cardiology and cardiac EP services continue to expand in India, population-specific normative anatomical data become increasingly relevant to procedural training, device design, and clinical guideline development. The present study represents a step toward addressing this data gap, providing quantitative CS measurements and TV morphological data derived from an Indian cadaveric cohort.

## Conclusions

This study demonstrates that meaningful anatomical variability exists in both CS length and TV morphology among specimens from the Indian population. The mean CS length is in accordance with internationally established normative ranges, yet the wide spread of individual values underscores the unpredictable nature of this structure in clinical practice. A sex-related trend toward greater CS length in male specimens was noted, consistent with general differences in cardiac dimensions between sexes, although formal statistical confirmation requires larger studies. The TV is present in the vast majority of individuals, with the semilunar form predominating; however, clinically challenging morphologies such as the cord-like and mesh-like variants collectively account for nearly one-third of TV-positive specimens.

Chi-square analysis confirmed that the TV morphological type distribution in the present Indian cohort does not differ significantly from that reported in the international literature, suggesting broad cross-population consistency in TV morphology patterns. This has reassuring implications for the generalizability of procedural guidance developed from non-Indian datasets to Indian clinical practice, pending confirmation in larger Indian studies. These anatomical findings are clearly relevant to the growing field of interventional cardiology in India, where population-specific data have historically been sparse. A thorough understanding of CS and TV anatomy enables proceduralists to anticipate technical obstacles, select appropriate instrumentation, and reduce procedural complication rates. The study encourages future larger-scale cadaveric and imaging-based investigations to consolidate normative anatomical data and explore the relationship between CS morphometry, donor sex and age, and procedural outcomes in the Indian population.
